# RegX3 Activates *whiB3* Under Acid Stress and Subverts Lysosomal Trafficking of *Mycobacterium tuberculosis* in a WhiB3-Dependent Manner

**DOI:** 10.3389/fmicb.2020.572433

**Published:** 2020-09-16

**Authors:** Amar Chandra Mahatha, Soumya Mal, Debayan Majumder, Sudipto Saha, Abhirupa Ghosh, Joyoti Basu, Manikuntala Kundu

**Affiliations:** ^1^Department of Chemistry, Bose Institute, Kolkata, India; ^2^Division of Bioinformatics, Bose Institute, Kolkata, India

**Keywords:** *Mycobacterium tuberculosis*, two-component systems, gene expression, acid stress, lysosomal trafficking, granuloma formation

## Abstract

Two-component systems (TCSs) are central to the ability of *Mycobacterium tuberculosis* to respond to stress. One such paired TCS is SenX3-RegX3, which responds to phosphate starvation. Here we show that RegX3 is required for *M. tuberculosis* to withstand low pH, one of the challenges encountered by the bacterium in the host environment, and that RegX3 activates the cytosolic redox sensor WhiB3 to launch an appropriate response to acid stress. We show that the *whiB3* promoter of *M. tuberculosis* harbors a RegX3 binding motif. Electrophoretic mobility shift assays (EMSAs) show that phosphorylated RegX3 (RegX3-P) (but not its unphosphorylated counterpart) binds to this motif, whereas a DNA binding mutant, RegX3 (K204A) fails to do so. Mutation of the putative RegX3 binding motif on the *whiB3* promoter, abrogates the binding of RegX3-P. The significance of this binding is established by demonstrating that the expression of *whiB3* is significantly attenuated under phosphate starvation or under acid stress in the *regX3*-inactivated mutant, *ΔregX3.* Green fluorescent protein (GFP)-based reporter assays further confirm the requirement of RegX3 for the activation of the *whiB3* promoter. The compromised survival of *ΔregX3* under acid stress and its increased trafficking to the lysosomal compartment are reversed upon complementation with either *regX3* or *whiB3*, suggesting that RegX3 exerts its effects in a WhiB3-dependent manner. Finally, using an *in vitro* granuloma model, we show that granuloma formation is compromised in the absence of *regX3*, but restored upon complementation with either *regX3* or *whiB3*. Our findings provide insight into an important role of RegX3 in the network that regulates the survival of *M. tuberculosis* under acid stress similar to that encountered in its intracellular niche. Our results argue strongly in favor of a role of the RegX3-WhiB3 axis in establishment of *M. tuberculosis* infection.

## Introduction

Tuberculosis remains a global health problem (World Health Organization, 2019, Global Tuberculosis Report). The causative agent *Mycobacterium tuberculosis* is endowed with the capacity to remain dormant for years within its host and to relapse under favorable conditions, including drug withdrawal. Efforts to contain the disease have been restricted by the absence of an efficacious vaccine, as well as the development of multi-drug resistance. The latter makes it important to search for new drug targets and approaches for chemotherapy, and specifically to understand how *M. tuberculosis* subverts the stress imposed by the host environment.

The success of *M. tuberculosis* rests with its ability to sense extreme conditions within its host such as nutrient deficiency, acid stress, hypoxia, and redox stress. Two-component systems (TCSs) are central to the ability of the bacterium to sense and respond to its environment (Tiwari et al., 2017). A membrane-bound sensor kinase (SK) and a cytosolic response regulator (RR) form two halves of canonical paired TCSs. Once the SK senses a stimulus, it is phosphorylated on a histidine residue. The phosphate is relayed on to an aspartate residue of the cognate RR, which regulates its binding to a distinct set of targets which make up its regulon. *M. tuberculosis* has twelve paired TCSs, six orphan RRs and two orphan SKs (Parish, 2014; Kundu, 2018). TCSs regulate gene expression to influence mycobacterial biofilm formation (Banerjee et al., 2019) virulence, pathogenesis, intracellular survival and the response to stress within its host (Bretl et al., 2011).

The TCS, SenX3-RegX3 is best characterized as a phosphate starvation responsive TCS which is required for intracellular survival and virulence of *M. tuberculosis* (Parish et al., 2003). Unlike many other TCSs, which are coordinately transcribed as operons, *senX3-regX3* contains a large intergenic region and the two genes can be differentially regulated. Thus, under nutrient starvation, there is bicistronic transcription of the operon, as opposed to phosphate depletion, which induces primarily monocistronic upregulation of *regX3* (Rifat and Karakousis, 2014). Additionally, phenotypic studies with mutants containing transposon disruptions in each gene have shown that RegX3 may function independently of its cognate SK, SenX3 (Rifat et al., 2014). RegX3 is inhibited when inorganic phosphate is abundant, in a manner requiring the phosphate-specific Pst transport system (Tischler et al., 2013). The function of the SenX3-RegX3 TCS of mycobacteria has been best studied in relation to phosphate starvation (Rifat et al., 2009; James et al., 2012). For example, it regulates protein secretion dependent on the ESX-5 secretion system (Elliott and Tischler, 2016) and membrane vesicle formation in an ESX-5 –independent manner (White et al., 2018). RegX3 is a regulator of the stringent response (Sanyal et al., 2013) and persister formation (Namugenyi et al., 2017). However, the other stress signals to which RegX3 is responsive, remain to be uncovered.

The seven WhiB proteins of *M. tuberculosis* are believed to be redox-sensing transcription factors (Larsson et al., 2012). WhiB3 is a virulence regulator of *M. tuberculosis* which senses host-generated nitric oxide (NO) and low levels of O_2_ (Steyn et al., 2002; Singh et al., 2007; Saini et al., 2012; Pacl et al., 2018). WhiB3 is required for survival of *M. tuberculosis* during reactive oxygen species (ROS) and reactive nitrogen species (RNS) stresses (Mehta and Singh, 2019) and during nutrient starvation. It is maximally induced at 2 weeks postinfection in the lungs of wild-type and immunodeficient (gamma interferon receptor-/-, Rag1-/-, and tumor necrosis factor alpha-/-) mice just prior to reaching a peak bacterial burden in the lungs, possibly indicating a role for this gene in quorum sensing *in vivo* (Banaiee et al., 2006). A *whiB3* mutant shows altered colony morphology and growth properties (Steyn et al., 2002; Singh et al., 2007). It modulates virulence lipids including phthioceroldimycocerosate (PDIM), polyacyltrehalose (PAT), sulfolipid (SL-1); and triacylglycerol (Singh et al., 2009). These polyketide lipids in turn, arrest host cell cycle at the G1/S transition (Cumming et al., 2017). The transcription of *whiB3* is significantly enhanced under acidic pH (Geiman et al., 2006) and WhiB3 is required to resist acid stress during infection (Mehta et al., 2016). Till date, its transcriptional regulation is incompletely understood. In pathogenic mycobacteria, PhoPR directly activates *whiB3* expression in response to low pH (Feng et al., 2018). In addition, a recent study has shown that the nitrogen regulator GlnR directly activates the expression of *whiB3* in *Mycobacterium smegmatis* (You et al., 2019).

Here we uncover a novel role of RegX3 in regulating the *M. tuberculosis* stress response by demonstrating that the survival of a *regX3* mutant is compromised under acid stress. We show that RegX3 is activated under acid stress. We demonstrate conclusively that RegX3 activates WhiB3 by direct binding to its promoter and that *whiB3* expression under acid stress is compromised in the absence of RegX3. Further we present evidence that (a) *M. tuberculosis* subverts lysosomal trafficking in a RegX3-WhiB3 dependent manner, and (b) granuloma formation in an *in vitro* model of infection of human PBMCs by *M. tuberculosis*, is regulated by RegX3 in a WhiB3-dependent manner. These observations argue in favor of a role of the RegX3/WhiB3 axis in facilitating *M. tuberculosis* infection.

## Materials and Methods

### Bacterial Strains and Growth Conditions

*Escherichia coli* Top10 and DH5α were used for cloning. *E. coli* BL21(DE3), and C41(DE3) were used for recombinant protein expression. *E. coli* was grown in Luria-Bertani (LB) Miller broth or on LB agar (Becton Dickinson, Difco) at 37°C. *M. tuberculosis* H37Rv or other genetically manipulated *M. tuberculosis* strains were grown in Middle Brook (MB) 7H9 (Difco) broth supplemented with 10% v/v ADC (Difco or Hi-Media Laboratories, India), 0.05% Tween 80 (Hi-Media Laboratories, India) and appropriate antibiotics where required, at 37°C with shaking. MB7H11 agar (Difco) supplemented with 10% v/v OADC (and antibiotics where required), was used as solid medium for plating *M. tuberculosis* strains. For *E. coli*, kanamycin sulfate (Roche Applied Science), ampicillin (Sigma), and hygromycin (Invitrogen) were used at concentrations of 50, 100, and 200 μg/ml, respectively. For *M. tuberculosis*, kanamycin and hygromycin were used at concentrations of 20 and 50 μg/ml, respectively. All experiments were performed following appropriate biosafety protocols approved by the Institutional Biosafety committee.

### Molecular Biology Procedures

Standard procedures were used for cloning and analysis of DNA, PCR, electroporation and transformation. The enzymes used to manipulate DNA were from Roche Applied Science, Fermentas and New England Biolabs.

### Generation of Genetically Manipulated Strains of *M. tuberculosis* H37Rv

*Mycobacterium tuberculosis* H37Rv lacking *regX3* (Δ*regX3*) was generated previously in our laboratory using temperature sensitive mycobacteriophages (Bardarov et al., 2002) as described by Banerjee et al. (2016). Briefly, approximately 800 bp flanking regions upstream and downstream of *regX3* were PCR amplified and cloned into pYUB854 at sites flanking the hygromycin cassette. Each construct was then packaged into temperature sensitive mycobacteriophages and delivered via infection into *M. tuberculosis*. Double crossovers (DCOs) were screened after 6 weeks by PCR and knockouts confirmed by Western blot using antibodies against RegX3. For complementation of *regX3*, a 2.5 kb region encompassing the *senX3-regX3* promoter, and the *senX3*-*regX3* ORFs were PCR amplified from genomic DNA and cloned into pMV306(Kan), a plasmid that allows integration at the *attB* site on the genome. A ∼900 bp senX3 region was deleted using Stu1 and the *regx3-*pMV306 construct was electroporated into the Δ*regX3* strain to obtain the *regX3* complemented strain (Δ*regX3* Comp.*regX3*), which was selected for growth on plates containing kanamycin as well as hygromycin.

For complementation with *whiB3*, the *whiB3* ORF was PCR amplified using sense and antisense primers 5′-ATGGTACCCATATGCCACAGCCGGAGCAGCTACCG 3′ and 5′ATTAAGCTTCTCGAGAGCTGTGCGGCGGATGCCGCG-3′, respectively, and cloned at the *Nde*I and *Xho*I sites of pet20b+ to generate a *whiB3* construct (pET20b *whiB3*-His) in frame with a C-terminal His tag. *whiB3* with a C-terminal His tag was then PCR amplified using the sense and antisense primers 5′ TTAGGATCCACCACAGCCGGAGCAGCTACCG3′and 5′ ATTAAGCTTGCTAGCTCAGTGGTGGTGGTGGTGGTGC-3′, respectively, and pET20b *whiB3*-His as template; and cloned at the *Bam*HI and *Hin*dIII sites of pMV261 (an *E. coli*-mycobacteria shuttle vector in which cloned genes are under the control of the *hsp60* promoter). This construct (pMV261 *whiB3*-His) was electroporated into the Δ*regX3* strain to obtain Δ*regX3* complemented with *whiB3* (Δ*regX3* Comp.*whiB3*), which was selected for growth on plates containing kanamycin as well as hygromycin.

### Growth of *M. tuberculosis* Under Phosphate Starvation

For phosphate starvation *M. tuberculosis* and its variants were grown in MB7H9 containing MOPS pH 6.6, 17.6 mM Na_2_HPO_4_, 7.35 mM KH_2_PO_4_, and 0.05% Tween 80, till the O.D._600_ reached 0.6. The cells were then centrifuged and washed twice with phosphate-free MB7H9, resuspended in either phosphate containing or phosphate free medium and allowed to grow at 37°C at a shaking speed of 120 rpm for 72 h. Aliquots were removed and cells were stored in RNA Later (Qiagen) at −80°C for isolation of RNA.

### Cloning, Expression and Purification of Recombinant Proteins

*Escherichia coli* BL21(DE3) harboring *regX3* cloned in pET28a was grown at 37°C with shaking and induced with isopropyl β-D-thiogalactopyranoside (IPTG) (120 μM) as described by Sanyal et al. (2013). Cells were lysed by sonication and His-RegX3 was purified from the cell-free supernatant by chromatography on Ni^2+^-NTA agarose. RegX3 (K204A) has been described by Banerjee et al. (2016). A recombinant MalE–EnvZ construct (gift from Dr. M. Igo, University of California, Davis, Davis, CA, United States) was transformed in *E. coli* BL21(DE3) and used for expression and purification using amylose affinity chromatography as described by Sharma et al. (2015). Briefly, cells were grown and induced with IPTG (100 μM) at 16°C for 20 h. Cells were disrupted by freeze-thaw cycles in the presence of lysozyme and MalE–EnvZ was purified from the cell-free supernatant by amylose affinity chromatography (NEB). EnvZ was autophosphorylated in kinase buffer (50 mM Tris–HCl, pH 8, 50 mM KCl, and 50 mM MgCl_2_) containing 20 mM ATP for 15 min at 37°C.

### Bioinformatic Analyses

The upstream promoter sequence of *whiB3* (−500 to +100) was searched for an inverted repeat motif having the following features: a 6–8 bp sequence, followed by a 5 bp spacer, followed by a second 6–8 bp region which is complementary to, and the inverse of the first 6–8 bp sequence. In-house PERL script was used for this search.

### *In vitro* Transphosphorylation Reactions

RegX3 or its DNA binding mutant (K204A) was phosphorylated using phospho-EnvZ as the phosphate donor (Roberts et al., 2011). The reaction was carried out in kinase buffer with 2.5 μM phosphorylated EnvZ and His-RegX3. Phosphorylated RegX3 (RegX3-P) was used for electrophoretic mobility shift assays (EMSAs).

### Electrophoretic Mobility Shift Assays

Binding of phospho-RegX3 (or its mutant) to the *whiB3* promoter was analyzed by EMSA. A *whiB3* promoter region (−307 to −157), containing the RegX3 binding sites was PCR amplified using the sense and antisense primer pair 5′-CAGCTTTCTTTGCGCTAATTTAGG-3′ (A) and 5′-CAATATCGGACCGTTGCGTGAG-3′ (C), respectively (Supplementary Figure S1). The antisense primer was Cy5-labeled. Mutant DNA was generated by overlap extension PCR (Supplementary Figure S1). The Cy5 labeled DNA (30 ng) was incubated with varying concentrations of phospho-RegX3 (or its mutant) in 4 mM Tris–HCl pH 8.0, containing 4 mM MgCl_2_, 5% (v/v) glycerol, 40 mM NaCl, 0.5 μg salmon sperm DNA, for 30 min at room temperature (RT). The reaction mix was run on a 6% TBE polyacrylamide gel and the DNA-protein complex was visualized using a Typhoon Trio Plus Imager (GE Healthcare). In other assays, EMSAs were carried out with mutated fragments of DNA, and the DNA-protein complexes were visualized using SYBR GOLD staining.

### Measurement of Promoter Activity

A DNA fragment encompassing the region –832 to +102 of *whiB3* was PCR-amplified using the primer pair 5′-TTAGGATCCCCACCGCCGACGCACCGC-3′ (sense) and 5′-CGGGGTACCCGTCGGGATGGAAGAACATCG-3′ (antisense) and cloned between the *Bam*HI and *Kpn*I restriction sites of pFPV27. The resulting construct or pFPV27 was electroporated separately into wild type *M. tuberculosis* and *ΔregX3* strains. Green fluorescent protein (GFP) fluorescence was measured in a microplate reader (Victor 1420 Multilabel counter) with excitation at 488 nm and emission at 535 nm.

### Chromatin Immunoprecipitation (ChIP)

Chromatin Immunoprecipitation (ChIP) was performed as described by Sharma et al. (2015). Briefly, growing *M. tuberculosis* cells were crosslinked using 1% formaldehyde followed by quenching with 250 μM glycine. The cells were lysed and DNA was sheared on a BIORUPTOR PLUS (Diagenode) with 25 cycles of 30 s/90 s on/off, respectively, so as to generate fragment sizes of approximately 250 bp. Immunoprecipitation (IP) was carried out using RegX3 antibody (raised by Thermo Fisher Scientific) and DNA was purified after decrosslinking with Proteinase K. Amplification of the *whiB3* promoter region was quantitated by ChIP-qPCR using the primer pair 5′-CAGCTTTCTTTGCGCTAATTTAGG-3′ (sense) and 5′-CAATATCGGACCGTTGCGTGAG-3′ (antisense).

### RNA Isolation From Intracellular Bacteria and qRT-PCR

RNA was prepared from intracellular *M. tuberculosis* following the method of Rohde et al. (2007) as described by Banerjee et al. (2019). Briefly, RAW264.7 cells were infected at a multiplicity of infection (MOI) of 10 for 4 h followed by treatment with gentamicin for another 2 h to remove extracellular bacteria. The cells were then washed and lysed in guanidine thiocyanate (GTC) buffer containing N-lauryl sarcosine, sodium citrate, and β-mercaptoethanol. Bacteria were pelleted, lysed using lysozyme and Trizol, followed by bead beating. RNA was prepared from the lysate after centrifugation using the Qiagen RNeasy Kit following the manufacturer’s protocol. RNA was treated with Turbo DNAse (Ambion). cDNA was synthesized using the cDNA synthesis kit (Thermo Fisher Scientific) according to the manufacturer’s instructions. For PCR, *whiB3* was amplified using sense and antisense primers 5′-AACGCAGACATCTGGAACTG-3′ and 5′-GGTGCCCTTGAGGAGTAGGT-3′, respectively. *regX3* was amplified using the sense and antisense primers 5′-CAG CGTTCCGGTGATCATG-3′ and 5′-CAGGCCGACCACCTT GTC-3′, respectively. 16s rRNA was amplified using the sense and antisense primers 5′-TCCCGGGCCTTGTACACA-3′ and 5′-CCACTGGCTTCGGGTGTTA-3′, respectively.

The quantitative PCR assay was performed with KAPA SYBR^®^ FAST Universal Q-PCR Mix (Kapa Biosystems Pty Ltd., Cape Town, South Africa). Melting curve analyses were run after each assay to check PCR specificity. Three serial dilutions were used for each cDNA. PCR was performed in triplicate for each dilution. The relative expression of the target gene was normalized to 16s rRNA The comparative C_T_ (also known as 2^–ΔΔ^C_T_) method was used for analyzing gene expression, with the assumptions that the difference of one cycle is two-fold and the PCR efficiency of the target gene is similar to that of the internal control (16s rRNA) (Livak and Schmittgen, 2001).

### Immunofluorescence Microscopy

RAW264.7 cells were grown on coverslips and infected with fluorescein isothiocyanate (FITC)-labeled *M. tuberculosis* strains as described by Banerjee et al. (2019). Cells were fixed with 4% (v/v) paraformaldehyde for 10 min permeabilized with 0.01% Triton X-100 in PBS, then treated with 2% BSA in PBS, followed by treatment with anti lysosomal-associated membrane protein 1 (LAMP1) antibody (Abcam) (1:500) overnight at 4°C. Cells were treated with Alexa 546-conjugated goat anti-rabbit antibody (Abcam) and coverslips were mounted with SlowFade (Thermo Scientific). Nuclei were stained with 4′,6-diamidino-2-phenylindole (DAPI). Slides were imaged by confocal microscopy.

### Acid Stress

Fifty milliliter of starter cultures of *M. tuberculosis* strains were grown in MB 7H9 broth to early log phase (O.D._600_ of 0.3), split into two aliquots and resuspended at pH 5.5 or pH 6.6. For pH 5.5, medium containing 0.02% Tyloxapol and 0.085% NaCl was buffered using 100 mM 2-(N-morpholino)ethanesulfonic acid (MES) (Healy et al., 2016). CFUs were followed over a period of 6 days and the percent reduction in CFUs of cultures grown at pH 5.5 relative to those grown at pH 6.6, was plotted. RNA was isolated from cultures exposed to different pH for different periods of time. Gene expression was evaluated by qRT-PCR using SYBR Green (Kappa Biosystems). The results were normalized relative to 16S rRNA levels.

### Granuloma Formation *in vitro*

Human peripheral blood mononuclear cells (PBMCs) were used for *in vitro* granuloma formation as described by Mehta and Singh (2019). Briefly, 5–10 ml blood was collected from a healthy human volunteer, diluted 1:1 and layered on Ficoll Paque (GE Healthcare) followed by centrifugation at 480 × *g* for 40 min at 18°C. The buffy coat containing monocytes was collected and washed twice in sterile cold KRG buffer (120 mM NaCl, 5 mM KCl, 1.5 mM MgCl_2_, 8.5 mM Na_2_HPO_4_, 1.7 mM NaH_2_PO_4_, and 10 mM dextrose). Cells were resuspended in complete RPMI medium with glutamine. The monocyte containing suspension was plated at a density of 1.5 × 10^6^ cells/ml in complete RPMI medium in 24 well plates and kept at 37°C in a CO_2_ incubator for 2 h. Single cell suspensions of each *M. tuberculosis* strain were made as described by Karim et al. (2011) and added at the required MOI to each well of the 24-well plate containing the PBMCs, centrifuged at 700 rpm for 5 min and incubated at 37°C in a CO_2_ incubator. After 4 h, 10 μg/ml gentamicin was added to remove the extracellular bacteria. After another 2 h, cells were washed thrice and complete RPMI medium was added to each well. At day 9 post-infection, cells were processed for Giemsa staining.

### Ethics Statement

All experiments with human samples were performed with the approval of the Institutional Human Ethics Committee (BIHEC/2017-18/1) and with consent from all donors (healthy volunteers in the laboratory).

### Giemsa Staining

Plates were centrifuged and each well was fixed with methanol for 5 min at RT. The fixative was removed, Giemsa stain was added and kept for 30 min at RT. Plates were washed with water to remove excess Giemsa stain. Images were taken in a Zeiss light Microscope with a 10 x objective.

### Statistical Analysis

Normality test was performed using GraphPad Prism v5 (applying D’Agostino-Pearson omnibus test and/or Shapiro–Wilk test). All the data distributed normally as inferred by the normality test. Student’s *t* test was performed for pairwise comparison. In the case of multigroup comparisons, analysis of variance (ANOVA) test was performed. For Anova, Tukey *Post hoc* test was used to confirm the significant differences between two groups. All analyses were performed using GraphPad Prism v5. A *p* value ≤ 0.05 was considered to be significant.

## Results

### RegX3 Is Required for the Survival of *M. tuberculosis* Under Acid Stress

**FIGURE 1:**
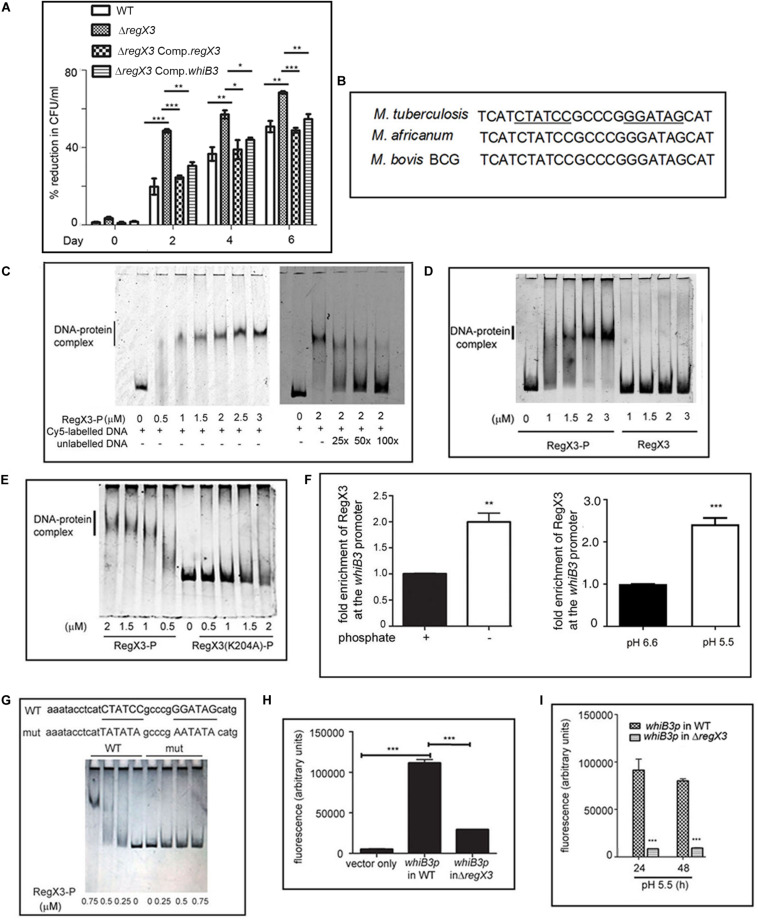
RegX3 is required for the survival of *M. tuberculosis* under acid stress and it binds to the promoter of *whiB3*. **(A)** Wild type *M. tuberculosis* or its genetically manipulated variants was grown at pH 5.5 or at pH 6.6 for different periods of time. Percent reduction in growth [as determined by enumerating colony forming units (CFUs)] of *M. tuberculosis* growth at pH 5.5 relative to growth at pH 6.6 is shown. **(B)** Sequence conservation of RegX3 binding motif of the *whiB3* promoter (*whiB3p*) in *M. tuberculosis*, *M. africanum*, and *M. bovis* BCG. **(C,D)** EMSA was performed by incubating a Cy5-labeled PCR fragment derived from *whiB3p* with different concentrations of phosphorylated RegX3 (RegX3-P) (left panel of **C**). Competitive EMSA was performed by incubating RegX3-P with Cy5-labeled DNA in the presence of increasing concentrations of unlabeled DNA (right panel of **C**) **(D)** EMSA was performed with phosphorylated or non-phosphorylated RegX3. **(E)** EMSA was performed with the DNA binding defective mutant (RegX3 K204A). DNA-protein complexes were visualized under a Typhoon biomolecular imager or by staining with SYBR GOLD. **(F)** ChIP analysis of RegX3 binding to *whiB3p*. *M. tuberculosis* was grown in phosphate enriched (+) or depleted (–) medium **(left panel)** or grown at different pH **(right panel)** and subjected to ChIP with RegX3 antibody. The association of RegX3 at the promoter of *whiB3* was quantitated by q-PCR of immunoprecipitated DNA or the input (as control) using primers specific for *whiB3p* or 16s rRNA. The fold enrichment with respect to control [i.e., bacteria grown in phosphate enriched medium **(left panel)**, or at pH 6.6 **(right panel)**] was set as 1. Data represent means ± S.D., *n* = 3. **(G)** EMSA was carried out by incubating wild type DNA or DNA mutated in the RegX3 box, with different concentrations of RegX3-P. Bands were visualized by SYBR GOLD staining. **(H,I)** *M. tuberculosis* (WT or Δ*regX3*) harboring either vector alone or *whiB3p* were grown either under phosphate stress **(H)** or acid stress **(I)** GFP fluorescence was measured in a fluorescence microplate reader. The data are represented as means ± SD, *n* = 3. ***p < 0.0001; **p < 0.001; *p < 0.05.

*M. tuberculosis* encounters an acidic environment in the phagosomes, and must wire its gene expression program to facilitate growth and persistence in this acidic environment. It is therefore important to understand the repertoire of regulators that enable *M. tuberculosis* to sense acid stress and to mount an appropriate response. Considering the importance of TCSs in sensing of the extracellular milieu, we searched the literature for regulators that are activated under acid stress or in the intraphagosomal milieu. PhoPR has been documented to be upregulated during acid stress (Bansal et al., 2017). The report that RegX3 is upregulated early during infection of macrophages by *M. tuberculosis* (Rohde et al., 2007), provided the cue for testing a probable role of RegX3 in mounting a response to acid stress. We tested and validated the upregulation of *regX3* following exposure of *M. tuberculosis* to acid stress (Table 1). Next we tested the survival of the wild type and the mutant *ΔregX3* previously reported by us (Banerjee et al., 2016) following exposure to either pH 6.6 or pH 5.5. Survival was monitored at different time intervals post-exposure by enumerating CFUs. There was a decrease in CFUs in wild type *M. tuberculosis* grown at pH 5.5 compared to cells grown at pH 6.6. However, the reduction in CFUs at pH 5.5 (compared to pH 6.6) was significantly higher in the absence of *regX3* (Figure 1A), suggesting that *regX3* contributes to the ability of the bacterium to withstand acid pH. This was confirmed by the observation that survival under acid stress was partially restored in the *regX3* complemented strain (*ΔregX3* Comp.*regX3*) (Figure 1A). Importantly, there were no significant differences in CFUs among the four strains on day 0 (i.e., at the start of the experiment).

**TABLE 1:** Relative fold changes of *regX3* in *M. tuberculosis* subjected to acid stress for different periods of time.

**Hours of acid stress**	**Fold-change**
2	1.52 ± 0.04
24	4.13 ± 0.32
48	2.62 ± 0.21

*Total RNA was isolated from *M. tuberculosis* before and after acid stress. Transcript abundance was determined by qRT-PCR. Comparisons were made with cells that had not been subjected to acid stress.*

We then searched for possible binding of RegX3 to the promoters of genes involved in regulating the response of *M. tuberculosis* to acid stress. WhiB3 is one such regulator. Its deletion lowers the survival of *M. tuberculosis* under acidic conditions (Mehta et al., 2016). We fell back on the report of Rustad et al. (2014) documenting that overexpression of RegX3 transcriptionally upregulates *whiB3*, therefore suggesting its role in regulating *whiB3* expression. For the purpose of this communication, we have focused on validating the regulation of WhiB3 by RegX3 under acid stress, and understanding its implications. As expected, the transcription of *whiB3* was enhanced when wild type *M. tuberculosis* was subjected to acid stress (Supplementary Table S1). The acid-induced upregulation of *whiB3* was abrogated in *ΔregX3* and restored upon complementation of the mutant with *regX3* (*ΔregX3* Comp.*regX3*) (Supplementary Table S1), suggesting that RegX3 is required for the transcription of *whiB3* under acidic conditions. The importance of a *regX3-whiB3* axis in regulating the survival of *M. tuberculosis* under acidic conditions, was tested by complementing *ΔregX3* with *whiB3* and assessing CFUs under acidic conditions. The observation that complementation with *whiB3* could partly restore the ability of *ΔregX3* to withstand acid stress (Figure 1A), argued in favor of a role of the *regX3-whiB3* axis in the survival of *M. tuberculosis* under acid stress.

An inverted repeat (GTGAAC) separated by five to seven unconserved nucleotides constitutes the RegX3 binding motif (Glover et al., 2007). For example, this motif has been identified on the *M. tuberculosis* polyphosphate kinase 1 (*ppk1*) promoter in our laboratory (Sanyal et al., 2013). An *in silico* analysis showed that a putative RegX3-binding motif is present in the *M. tuberculosis whiB*3 promoter region (Figure 1B). This RegX3 binding palindrome was conserved in *Mycobacterium africanum* and *Mycobacterium bovis* BCG, two members of the *M. tuberculosis* complex. It was absent in *Mycobacterium leprae* or the fast-growing *M. smegmatis*.

### RegX3 Binds to the Promoter of *whiB3* and Regulates *whiB3* Promoter Activity

In order to test the binding of RegX3 to the *whiB3* promoter region, we amplified the region –307 to −157 of the *whiB3* promoter to generate a Cy5-labeled DNA and used this for EMSAs. RegX3-P bound to the aforesaid DNA in a concentration-dependent manner (Figure 1C, left panel). This binding could be competed by unlabeled DNA, suggesting the specificity of the binding (Figure 1C, right panel). Phosphorylation of RegX3 is usually required for optimal DNA binding activity (Sanyal et al., 2013). In line with this, unphosphorylated RegX3 was not able to bind the *whiB3* promoter-derived DNA (Figure 1D), suggesting that phosphorylation is required for binding of RegX3 to the *whiB3* promoter. In previous studies, we have shown that amino acid lysine 204 (K204) is required for DNA-binding activity of RegX3 (Banerjee et al., 2016). RegX3 (K204A) was unable to bind the *whiB3* promoter fragment (Figure 1E) confirming the specificity of the binding.

In order to analyze the physiological relevance of the aforesaid binding, we performed ChIP assays under conditions associated with upregulation of RegX3. RegX3 is best characterized as a phosphate starvation-responsive RR (Rifat et al., 2009). We therefore subjected cells to phosphate starvation and tested the association of RegX3 with the *whiB3* promoter region, using an antibody to RegX3. The binding of RegX3 to the *whiB3* promoter region was confirmed by ChIP-qPCR (Figure 1F, left panel), arguing in favor of a physiological role of RegX3 in regulating *whiB3* expression under stress. In order to test the role of RegX3 in regulating *whiB3* under acid stress, we further performed ChIP-qPCR after subjecting cells to acid stress. The association of RegX3 with the *whiB3* promoter region was enhanced in cells grown at pH 5.5 compared to cells grown at pH 6.6 (Figure 1F, right panel).

Finally, we confirmed the role of the putative RegX3 binding palindrome identified on the *whiB3* promoter by mutational analysis. The sequences CTATCC and GGATAG in the palindrome were substituted with the sequences, TATATA and AATATA, respectively (Figure 1G) to generate a mutant DNA. RegX3 failed to bind to this mutated DNA (Figure 1G), confirming that this region is required for the binding of RegX3 to the *whiB3* promoter. To further elucidate the requirement of RegX3 in the expression of *whiB3*, the *whiB3* promoter was cloned in a promoterless GFP vector pFPV27, electroporated in *M. tuberculosis* (or *ΔregX3*) and GFP activity was monitored under phosphate starvation (a known trigger for RegX3 expression) or acid stress. Phosphate starvation activated the *whiB3* promoter (Figure 1H). However, this was significantly diminished in *ΔregX3* (Figure 1H). Promoter activity was also observed in cells exposed to acid stress (Figure 1I), and was significantly diminished in *ΔregX3* (Figure 1I).

### Intracellular *whiB3* Expression Is Regulated by RegX3

We next addressed the question whether the regulation of *whiB3* by RegX3 is relevant within the intracellular milieu of the host. It has been reported that *whiB3* is upregulated early during infection of macrophages by *M. tuberculosis* (Rohde et al., 2007). We therefore analyzed the expression of *whiB3* during infection of macrophages by either wild type or *regX3*-inactivated *M. tuberculosis*. Expression of *whiB3* was compromised in the absence of *regX3* compared to the wild type (Table 2) and restored upon complementation with *regX3*. These observations suggested that RegX3 regulates the expression of *whiB3* of *M. tuberculosis* within the intracellular milieu of its host.

**TABLE 2:** Relative fold changes of *whiB3* in different *M. tuberculosis* strains grown in macrophages.

**Strain**	**Fold change**
*ΔregX3*	0.49 ± 0.02
Δ*regX3* Comp.*regX3*	0.80 ± 0.08

*Different strains of *M. tuberculosis* were grown in macrophages. Cells were lysed, bacteria were harvested and total bacterial RNA was isolated. Transcript abundance was determined by qRT-PCR. The relative fold changes of the different mutants of *M. tuberculosis* were compared with that of wild type *M. tuberculosis* (set as 1) grown in macrophages.*

### RegX3 Regulates Trafficking of *M. tuberculosis* to Lysosomes in a WhiB3-Dependent Manner

**FIGURE 2:**
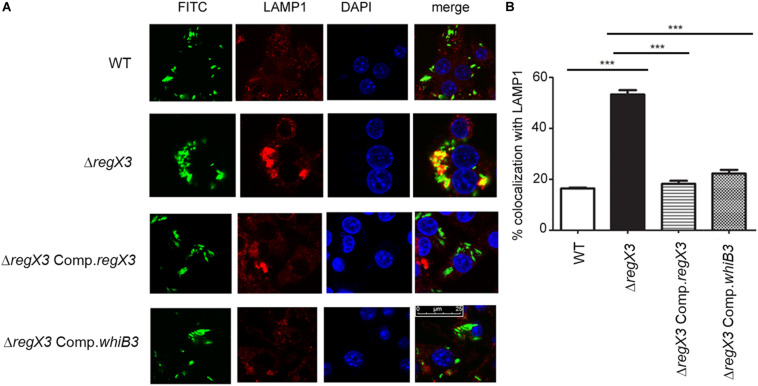
RegX3 and WhiB3 are required for trafficking of *M. tuberculosis* to lysosomes **(A)** RAW264.7 cells were infected with FITC-labeled (green) *M. tuberculosis* (WT, Δ*regX3*, Δ*regX3* Comp*.regX3* or Δ*regX3* Comp.*whiB3*), fixed and immunolabeled with anti-LAMP1 followed by Alexa 546-conjugated secondary antibody (red). Cells were stained with DAPI to visualize the nuclei. Colocalization of green and red fluorescence indicates bacterial trafficking to the lysosomal compartment. **(B)** Percent colocalization of each of the four strains with LAMP1 was calculated by counting at least 100 bacteria from five different fields. Data represents means ± S.D., three independent experiments. ***p < 0.001.

Intracellular trafficking to lysosomes reduces mycobacterial burden in macrophages, and virulent mycobacteria have the ability to evade trafficking to macrophages. *ΔregX3* showed reduced survival in macrophages compared to the wild type (Banerjee et al., 2016). We therefore tested whether the ability of *M. tuberculosis* to evade trafficking to lysosomes is compromised in the absence of *regX3.* Following infection, the colocalization of FITC-labeled *M. tuberculosis* with the lysosomal marker LAMP1 was enumerated by fluorescence microscopy. The association of *ΔregX3* with lysosomes, was higher than that of wild type *M. tuberculosis* (Figures 2A,B). This was partly reversed in *ΔregX3* Comp.*regX3* (Figures 2A,B). Interestingly, complementation with *whiB3* (*ΔregX3* Comp.*whiB3*) was equally effective in reversing the trafficking of *M. tuberculosis* to lysosomes. These results suggested that RegX3 subverts the trafficking of *M. tuberculosis* to lysosomes in a WhiB3-dependent manner.

### RegX3 Has a Role in Granuloma Formation in *M. tuberculosis*

**FIGURE 3:**
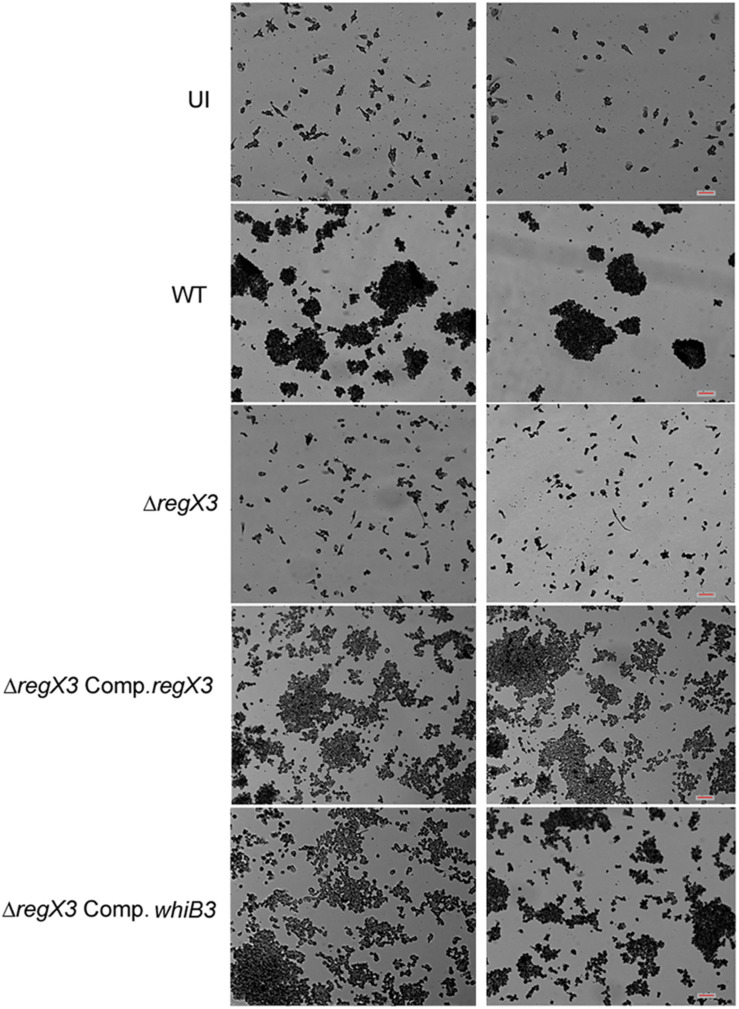
*In vitro* granuloma formation after infection of PBMCs with different strains of *M*. *tuberculosis.* One million human PBMCs were infected with different strains of *M. tuberculosis* at an MOI of 0.01; or were left uninfected (UI) and stained with May-Grünwald Giemsa stain at day 9 post-infection. Images were taken at 10× magnification. Each **left** and **right panel** shows two different fields out of at least five fields captured. Scale bar denotes 100 μm.

Human PBMCs have been previously used to elicit granuloma formation *in vitro* (Puissegur et al., 2004; Guirado et al., 2015). Previous reports have shown that WhiB3 is required for granuloma formation *in vitro* (Mehta and Singh, 2019). Considering that *whiB3* expression is regulated by RegX3, we tested the role of RegX3 in granuloma formation *in vitro* 1 million PBMCs were infected with *M. tuberculosis* at an MOI of 0.01. Aggregates of PBMCs (>100 μm) were observed in the case of infection with the wild type bacterium. However, there was a striking absence of these aggregates when PBMCs were infected with *ΔregX3* (Figure 3). Complementation of *ΔregX3* with *regX3* (*ΔregX3* Comp.*regX3*) restored the ability of the bacterium to elicit aggregation and granuloma formation (Figure 3). Interestingly, *ΔregX3* Comp.*whiB3* was also able to elicit granuloma formation (Figure 3). These results strongly suggested that the role of RegX3 in granuloma formation is dependent on WhiB3.

## Discussion

The TCSs play an important role in regulating the ability of *M. tuberculosis* to respond to the stressful conditions it encounters during the course of establishing a successful infection. Among the paired TCSs, SenX3-RegX3 is best characterized as a phosphate starvation responsive TCS (Rifat et al., 2009). The other stress signals that it responds to, remain unclear. Its regulon is also incompletely characterized. The intracellular niche for *M*. *tuberculosis* is the macrophage, where survival under low pH poses a challenge. We searched for regulators that are activated when *M. tuberculosis* resides in the intracellular milieu of the macrophage, where the pH is acidic. RegX3 is one such regulator (Rohde et al., 2007). *ΔregX3* is compromised in its ability to survive in macrophages compared to the wild type (Banerjee et al., 2016). In the present study we show that RegX3 is upregulated under acid stress (Table 1) and that it is required for the survival of *M. tuberculosis* under acid stress (Figure 1A).

The 4Fe-4S protein WhiB3 is activated under acid stress (Geiman et al., 2006) and in the response to vitamin C, a trigger of dormancy (Nandi et al., 2019). WhiB3 is required for the induction of immunomodulatory lipids and for blocking phagosomal maturation (Pacl et al., 2018). We show for the first time that RegX3 regulates *whiB3* under both phosphate starvation and acid stress.

Previous studies have shown that the *whiB3* promoter regions from *M. tuberculosis* and *Mycobacterium marinum* contain a PhoP box and that PhoP regulates *whiB3* under acid stress (Feng et al., 2018), specifically in slow-growing mycobacteria such as *M. tuberculosis.* In this study we have identified a RegX3-binding palindrome upstream of *whiB3* in *M. tuberculosis* (Figure 1B). The PhoP binding site on the *whiB3* promoter is located between −310 and −271 of the *whiB3* upstream region (Feng et al., 2018). The RegX3 binding site is located between −244 and −227. The RegX3 binding sequence is conserved in two members of the *M. tuberculosis* complex, namely *M. africanum* and *M. bovis* BCG. This sequence is not conserved in other mycobacteria such as *M*. *leprae* or the fast-growing *M. smegmatis*. Unlike the PhoP box, the RegX3 box is also absent in *Mycobacterium kansasii* and *M. marinum*, suggesting that dual regulation of *whiB3* by PhoP and RegX3 under acid stress, may be unique to the *M. tuberculosis* complex. This suggests that a complex regulation of *whiB3* by both PhoP and RegX3 is likely tailored to aid the survival of *M. tuberculosis* within its intracellular niche.

We observed that RegX3 binds to the *whiB3* promoter in a phosphorylation-dependent manner (Figure 1D), and that a DNA-binding mutant of RegX3 fails to do so (Figure 1E), confirming the specificity of the binding. Mutation of a set of nucleotides within the putative RegX3-binding palindrome, abrogated RegX3 binding (Figure 1G), confirming the requirement of the palindromic sequence in the *whiB3* promoter for RegX3 binding. We also confirmed binding of RegX3 to the *whiB3* promoter region by ChIP using RegX3-specific antibody (Figure 1F). The *regX3*-deleted strain, Δ*regX3*, failed to activate the *whiB3* promoter and was compromised in its ability to survive under acid stress. It showed compromised levels of *whiB3* expression under either phosphate starvation (a known signal for RegX3 activation) or acid stress, suggesting the likely physiological relevance of the RegX3-WhiB3 axis. Taken together, these findings confirmed that RegX3 is a regulator of *whiB3* in *M. tuberculosis*.

During infection, *M. tuberculosis* colonizes macrophages. We have previously shown that Δ*regX3* is compromised in terms of its ability to survive in macrophages (Banerjee et al., 2016). In line with this, it showed increased trafficking to the lysosomal compartment (Figure 2). Concanamycin A, an inhibitor of vacuolar ATPases, blocks acidification of the phagosome in macrophages infected with *M. tuberculosis* (Rohde et al., 2007). In these conditions, intracellular *whiB3* induction is inhibited, suggesting that acidification of the macrophage, triggers *whiB3* induction, and that *whiB3* probably has a role in subverting the trafficking of *M. tuberculosis* to macrophages. Interestingly, trafficking of *ΔregX3* to the lysosomal compartment was reversed upon complementation with either *regX3* or *whiB3*. These observations suggested that RegX3 functions at acid pH and in macrophages, in a WhiB3-dependent manner.

*whiB3* is reportedly required for *M. tuberculosis* survival under acidic stress (Mehta et al., 2016). Since the RegX3 binding box as well as the PhoP box have been identified only in the *whiB3* promoters of *M*. *tuberculosis* complex members, we suggest that the PhoPR-*whiB3* and the RegX3-*whiB3* regulatory pathways may act in concert in a manner unique to the *M. tuberculosis* complex, facilitating successful infection. In future studies, it would be of interest to evaluate the status of the PhoP and the RegX3 boxes in the *whiB3* promoters of clinical strains of *M*. *tuberculosis*, and any possible link they may have with mycobacterial virulence. It would also be of importance to dissect the hierarchy of functioning of PhoP and RegX3 in the regulation of *whiB3*.

**FIGURE 4:**
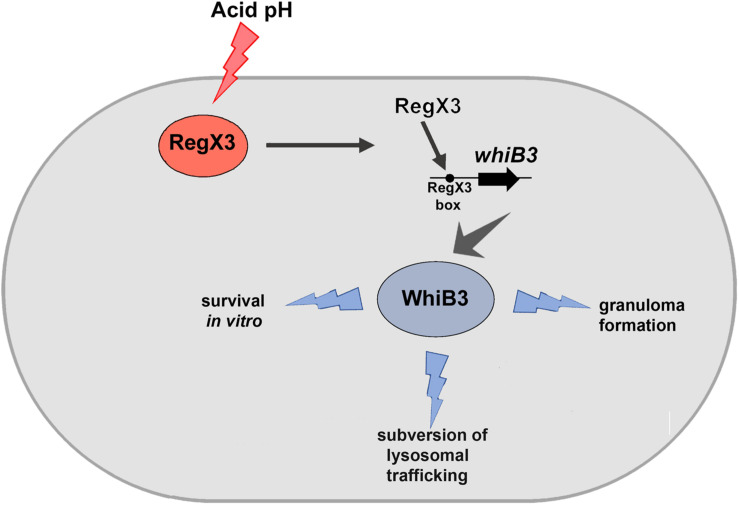
Schematic representation of the effect of RegX3 induction under acid stress in *M*. *tuberculosis*. RegX3 is induced upon exposure of *M. tuberculosis* to acid pH. It binds to the RegX3 box on the *whiB3* promoter to trigger *whiB3* induction. WhiB3 subsequently mediates subversion of lysosomal trafficking of *M. tuberculosis* to macrophages and granuloma formation *in vitro.*

*M. tuberculosis* resides in granulomas within its host. Previous studies have shown that there is reduced lung inflammation in mice infected with *ΔwhiB3* compared to wild type *M. tuberculosis*. There is scant knowledge of the *M*. *tuberculosis* factors that facilitate granuloma formation. Considering the physiological differences between animals and humans with respect to *M*. *tuberculosis* infection, we chose to study the role of the RegX3-WhiB3 axis in a human *in vitro* granuloma model. Here we show that granuloma formation in an *in vitro* model (i.e., the formation of aggregates >100 μm), is compromised in the absence of RegX3 (Figure 3). Interestingly granuloma formation is restored upon complementation of Δ*regX3* with either *regX3* or with *whiB3*, underscoring the importance of the RegX3-WhiB3 axis in granuloma formation, possibly through the WhiB3-dependent induction of immunomodulatory lipids. In summary, our present studies show that acid stress induces RegX3, which binds to the promoter of *whiB3* to trigger its expression. WhiB3 subsequently mediates subversion of lysosomal trafficking of the bacterium in macrophages, and granuloma formation *in vitro* (Figure 4). The enhancement of understanding of pathogen biology through the present study, should aid in designing intervention strategies in future. It would be of interest to compare lung pathology elicited *in vivo* by *M. tuberculosis ΔregX3* complemented with either *regX3* or *whiB3*, and to generate a more complete understanding of the genes regulated by the RegX3/WhiB3 axis within the host milieu.

## Data Availability Statement

The original contributions presented in the study are included in the article/Supplementary Material, further inquiries can be directed to the corresponding author.

## Ethics Statement

The studies involving human participants were reviewed and approved by the Institutional Human Ethics Committee (BIHEC/2017-18/1). The patients/participants provided their written informed consent to participate in this study.

## Author Contributions

JB and MK designed the research and analyzed the data. AM performed the experiments and analyzed the data. SM performed fluorescence microscopy. DM was involved in *in vitro* granuloma formation experiments. AG and SS performed the bioinformatic analyses. All authors contributed to the article and approved the submitted version.

## Conflict of Interest

The authors declare that the research was conducted in the absence of any commercial or financial relationships that could be construed as a potential conflict of interest.
