# Supplementary material for: RegX3 Activates whiB3 Under Acid Stress and Subverts Lysosomal Trafficking of Mycobacterium tuberculosis in a WhiB3-Dependent Manner
Source: Front Microbiol. 2020 Sep 16;11:572433. doi: 10.3389/fmicb.2020.572433 (PMC7525159; doi:10.3389/fmicb.2020.572433)
Supplement: Supplementary file 1 [file Data_Sheet_1.PDF]

Supplementary data

Title: RegX3 activates whiB3 under acid stress and subverts lysosomal trafficking of *Mycobacterium tuberculosis* in a WhiB3-dependent manner

Authors: Amar Chandra Mahatha, Soumya Mal, Debayan Majumder, Sudipto Saha, Abhirupa Ghosh, Joyoti Basu and Manikuntala Kundu

One table (Table S1) and one figure (Fig. S1)

Table S1. Relative fold changes of *whiB3* in different *M. tuberculosis* strains subjected to acid stress for 24 h

Hours of acid stress	Fold-change
WT	1.6 ± 0.17
ΔregX3	0.57 ± 0.03
ΔregX3 Comp.*regX3*	1.5 ± 0.14

Total RNA was isolated from different *M. tuberculosis* strains subjected to acid stress. Transcript abundance was determined by qRT-PCR. For each strain, RT-PCR signals of cells subjected to acid stress were compared to cells that had not been subjected to acid stress. Data are the means of three replicates.

IR1 IR2

5’ ....Tgggcaaatacctcat**ctatccgcccgggatag**catgcggcgcaggcggc...3’ (WT)

5’ ....Tgggcaaatacctcat**tatatagcccgaatata**catgcggcgcaggcggc...3’(MUTANT)

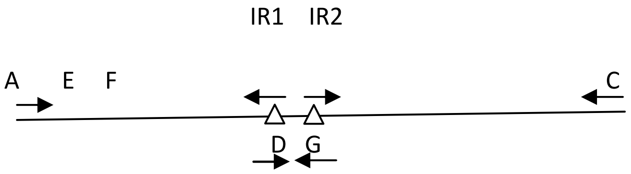

Primers A and C were used for PCR to generate the wild type (WT) *whiB3* promoter DNA.

For mutating IR1 (underscored), the initial rounds of PCR were performed with primers D, C and A, E to give fragments of 103 bp and 85 bp respectively. The products were used as templates for the final round of PCR using primers A and C. The product with substituted IR1 was used as template for generating substitutions in IR2 (underscored) as well. The initial rounds of PCR were performed with primers A,Gand F,C to give fragments of 91 bp and 92 bp. These fragments were used as template for the final round of PCR using primers A and C. The resultant 150 bp product (MUTANT) harbored substitutions in IR1 as well as IR2.

Sequences of primers:

5’ ---------------------------------------3’

A: CAGCTTTCTTTGCGCTAATTTAGG

C: CAATATCGGACCGTTGCGTGAG

D: TGGGCAAATACCTCATTATATAGCCCGGGATAGCATGC

E: GCATGCTATCCCGGGCTATATAATGAGGTATTTGCCCA

F: CTCATTATATAGCCCGAATATACATGCGGCGCAGGCGG

G: CCGCCTGCGCCGCATGTATATTCGGGCTATATAATGAG

Figure S1. Details of PCR reactions for the generation of substitution mutants of the putative RegX3 binding sites (IR1 and IR2) on the *whiB3* promoter.
